# Using Cu_2_O/ZnO as Two-Dimensional Hole/Electron Transport Nanolayers in Unleaded FASnI_3_ Perovskite Solar Cells

**DOI:** 10.3390/ma17051064

**Published:** 2024-02-26

**Authors:** Masood Mehrabian, Maryam Taleb-Abbasi, Omid Akhavan

**Affiliations:** 1 Department of Physics, Faculty of Basic Science, University of Maragheh, Maragheh P.O. Box 55181-83111, Iran; 2 Department of Physical Chemistry, Faculty of Chemistry, University of Tabriz, Tabriz P.O. Box 51666-16471, Iran; 3 Department of Physics, Sharif University of Technology, Tehran P.O. Box 11155-9161, Iran

**Keywords:** nanomaterials, zinc oxide, copper oxide, formamidinium Sn-based perovskites, SCAPS-1D solar simulator, Pb-free solar cells

## Abstract

A Pb-free FASnI_3_ perovskite solar cell improved by using Cu_2_O/ZnO as two-dimensional-based hole/electron transport nanolayers has been proposed and studied by using a SCAPS-1D solar simulator. To calibrate our study, at first, an FTO/ZnO/MAPbI_3_/Cu_2_O/Au multilayer device was simulated, and the numerical results (including a conversion efficiency of 6.06%, an open circuit potential of 0.76 V, a fill factor parameter of 64.91%, and a short circuit electric current density of 12.26 mA/cm^2^) were compared with the experimental results in the literature. Then, the conversion efficiency of the proposed FASnI_3_-based solar cell was found to improve to 7.83%. The depth profile energy levels, charge carrier concentrations, recombination rate of electron/hole pair, and the FASnI_3_ thickness-dependent solar cell efficiency were studied and compared with the results obtained for the MAPbI_3_-containing device (as a benchmark). Interestingly, the FASnI_3_ material required to obtain an optimized solar cell is one-half of the material required for an optimized MAPbI_3_-based device, with a thickness of 200 nm. These results indicate that developing more environmentally friendly perovskite solar cells is possible if suitable electron/hole transport layers are selected along with the upcoming Pb-free perovskite absorber layers.

## 1. Introduction

Recently, perovskite-based solar cells (PSCs) have attracted significant attention because of their features, including excellent extinction coefficient, solution-processed synthesize methods, suitable band gap engineering, and remarkable growth in power conversion efficiency (PCE) up to ~25% [1,2] (which was initially only 3.8% in 2009 [3]). The appropriate selection of the hole transport layer (HTL) and electron transport layer (ETL) in a perovskite solar cell is a vital factor in the enhancement of the stability and performance of PSCs [4,5]. The cost and efficiency of PSCs are usually determined by the kind of HTL that is employed. The HTL is a component that impacts the performance of PSCs by extracting the photo-induced holes from the perovskite region to the external electrode.

The most usual HTL used in PSCs is the spiro-OMeTAD, which, besides being very costly, faces various degradation issues. Therefore, it cannot be considered a proper choice for large-scale production. Hence, a low-cost and stable p-type metal oxide should be selected as the HTL in the next generation of PSCs. The optimum selection of metal oxide exhibits unique properties for electric charges to transport between layers by creating a cascade structure that is in better alignment with the energy bands in a solar cell. It was found that the application of metal oxides as effective charge transport layers can significantly improve the performance and stability of PSCs [6]. Several inorganic materials, including NiO, Cu_2_O, and CuO, have been introduced as acceptable candidates for application as HTLs [7,8,9]. Among these various materials, cuprous oxide (Cu_2_O) has been proposed as a superior HTL with a suitable band gap of ~2 eV [10], a high stability, and improved efficiency [11]. For example, recently, Cu_2_O was used as an HTL for a ZnO-based PCS fabricated at room temperature [12]. The fabricated device with an FTO/ZnO/CH_3_NH_3_PbI_3_/Cu_2_O/Au structure showed open circuit potential (V_OC_), short circuit electric current density (J_SC_), fill factor parameter (FF), and PCE values of 0.76 V, 12 mA/cm^2^, 63%, and 6.02%, respectively.

However, the presence of toxic lead metal in the structure of methylammonium Pb-iodide (MAPbI_3_) perovskite-based solar cells (as the material initiated the interest in lead halide perovskites for solar cell applications [13]) is a major concern from an environmental perspective. Therefore, introducing non-toxic elements into the perovskite structure is required. In this regard, different kinds of less toxic cations including Sn, Ge, Bi, Sb, and Ti could be substitute elements for lead in PSCs [14,15]. Among them, Sn-based halide perovskites have received more attention because of their similar properties to Pb-halide PSCs and comparable PCE values [16]. As a two-dimensional layer nanomaterial, graphene enhances the photoelectric conversion efficiency of dye-sensitized solar cells [17]. 

Recent investigations on formamidinium tin iodide (FASnI_3_) with the chemical formula of CH_4_N_2_SnI_3_ and a direct energy band gap of 1.41eV demonstrated that it can be considered a good candidate as an absorber layer in non-toxic PSCs. Moreover, FASnI_3_-based PSCs are distinguished as being more stable and efficient absorber layers than MASnI_3_-based ones due to their better thermal stability and greater band gap [18]. Concerning this, Neol et al. proposed a Pb-free tin halide CH_3_NH_3_SnI_3_-based solar cell, which showed a PCE value of 6% [19]. A Pb-free formamidinium tin-based PSC with optimized photovoltaic parameters of a V_oc_ value of 1.81 V, a J_sc_ value of 31.20 mA/cm^2^, an FF value of 33.72%, and a PCE of 19.08% was also examined by Kumar et al. [20]. Koh et al. studied FASnI_3_-based perovskite solar cells with an FTO/TiO_2_/FASnI_3_/spiro-OMeTAD/gold multilayer structure. The results showed J_SC_, V_OC_, FF, and PCE values of 12.4 mA/cm^2^, 0.26 V, 44%, and 1.41%, respectively [21]. Bian et al. found that the performance of Pb-free formamidinium Sn-triiodide PSC can be improved by Sn source purification, resulting in a maximum PCE of 6.75%, a V_OC_ of 0.58 V, a J_SC_ of 17.5 mA/cm^2^, and an FF of 66.3% in an indium tin oxide/PEDOT:PSS/CH_4_N_2_SnI_3_/C_60_/BCP/silver multilayer structure [22]. López-Fernández et al. provided a comprehensive summary of the recent advancements, persistent challenges, and prospects regarding the synthesis, optical spectroscopy, and optoelectronic devices of Pb-free halide perovskite thin films and nanocrystals. This endeavor aims to offer guidance to both current researchers in this domain and future entrants. In general, they demonstrated the potential of Pb-free perovskites for applications in photovoltaics and optoelectronics [23]. Ran et al. reported a perovskite precursor solution of FASnI_3_ utilized to incorporate conjugated large-volume amines, specifically 3-phenyl-2-propen-1-amine, thereby enhancing the photovoltaic performance of the resulting films. They also introduced a viable method to achieve equilibrium between stability and charge extraction in tin-based perovskites and underscored the significant prospects for enhancing efficiencies in tin-based PSCs [24]. A functional additive based on pseudohalides in FASnI_3_ was shown to be advantageous for enhancing the formation of the film, as well as for addressing imperfections both at the interface and within the bulk material, in a study by Dhruba B. Khadka et al. [25]. Youssef El Arfaoui et al. proposed and analyzed an optimized tandem solar device consisting of lead-free perovskite CsGeI_3_/FASnI_3_ with a high efficiency of 30.42%. This optimization was achieved through a numerical simulation using SCAPS [26].

However, there has not been an investigation concerning the performance of the FASnI_3_ absorber layer in a perovskite-based solar cell structure containing Cu_2_O/ZnO as a two-dimensional (2D)-based HTL/ETL. Compared to 1D nanomaterials, 2D nanostructures, such as nanosheets or nanoplates, with large exposed surface areas and specific crystal facets, undoubtedly offer more space for adsorption and electron transmission. This could be a possible approach to enhance the photovoltaic properties of ZnO-based solar cells.

Therefore, in the present study, an FTO/ZnO/FASnI_3_/Cu_2_O/Au multilayer structure is proposed and studied by using a SCAPS-1D solar simulator. The findings, such as the depth profile energy levels, charge carrier concentrations, electron/hole pair recombination rates, and the FASnI_3_ thickness-dependent solar cell efficiency, are compared with those obtained for the FTO/ZnO/MAPbI_3_/Cu_2_O/Au structure as benchmarks. 

## 2. Simulation Parameters

**Figure 1:**
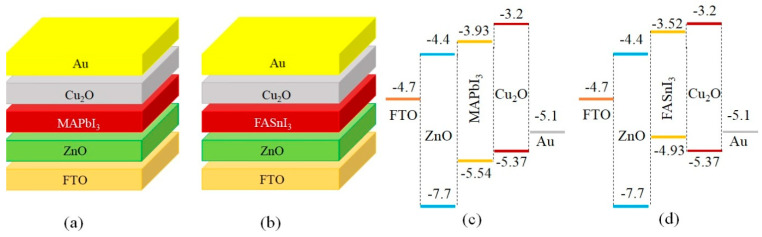
Architecture of the perovskite solar cells designed based on (**a**) the MAPbI_3_ and (**b**) the FASnI_3_ absorber layers and the energy level diagrams of (**c**) the MAPbI_3_ and (**d**) the FASnI_3_-based PSCs.

A simulation of planar FTO/ZnO/Perovskite/Cu_2_O/Au heterojunction solar cells was performed for both MAPbI_3_ and FASnI_3_ perovskites by utilizing the Solar Cell Capacitance Program (SCAPS-1D) [27,28]. The total thicknesses of the ZnO and Cu_2_O 2D multilayer structures were considered to be 50 (~95 monolayers) and 200 nm, respectively. These parameters were selected so that comparing the results with the experimental results reported in the literature was possible. The shallow donor and acceptor density in the ZnO and Cu_2_O nanolayers was set as 10^18^ cm^−3^ [28,29]. The solar cell architecture and corresponding energy level diagrams of the MAPbI_3_ and FASnI_3_-based PSC devices are presented in Figure 1. The basic parameters of layers which are based on some previously published works are listed in Table 1 [30,31,32,33,34,35]. 

**Figure 2:**
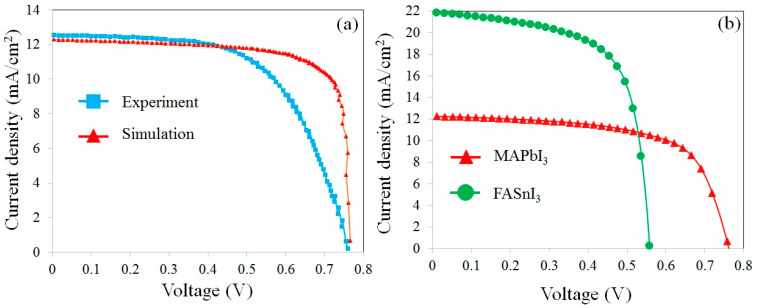
Comparison of the J-V curves obtained from (**a**) the experimental and simulation results for the FTO/ZnO/MAPbI_3_/Cu_2_O/Au solar cell and (**b**) the simulated results for the MAPbI_3_ and FASnI_3_-based solar cells.

Figure 2a exhibits the short circuit electric current density–voltage (J-V) curves of the experimental [12] and simulation results for solar cells with FTO/ZnO/MAPbI_3_/Cu_2_O/Au structures. A comparison between the photovoltaic parameters of the simulated device and the reported measurements is shown in Table 2. The consistency of the results for the MAPbI_3_-based structure provided a justification for the simulation method. Then, the justified model was extended to the new FASnI_3_-based cells for further evaluation. In Figure 2b, it is seen that the device with a MAPbI_3_ absorber has a larger V_OC_ but a smaller J_SC_ than the device with a FASnI_3_ layer. This decrease in the V_OC_ of a device with a FASnI_3_ absorber refers to the location of the highest occupied molecular orbital (HOMO) level of the absorber layer, which is higher than the valance band level of Cu_2_O. In such a situation, the extraction of holes becomes slower, which results in an increase in the electron/hole recombination rate at the perovskite/HTL interface. Therefore, the V_OC_ was reduced from 0.76 to 0.54 V. In fact, it can be seen in Figure 1c,d that the C.B level of MAPbI_3_ is located at −3.93 eV, while that of FASnI_3_ is located at −3.52 eV. Therefore, the difference between C.B of Cu_2_O and the absorber layer in the device with MAPbI_3_ is 0.73 eV, while it is 0.32 eV in the device with FASnI_3_. As a result, the recombination rate in the device with MAPbI_3_ is lower than that of the device with FASnI_3_. That is why the V_oc_ in the device with MAPbI_3_ (0.76 V) is higher than that of the device with FASnI_3_ (0.54 V).

## 3. Results and Discussion

**Figure 3:**
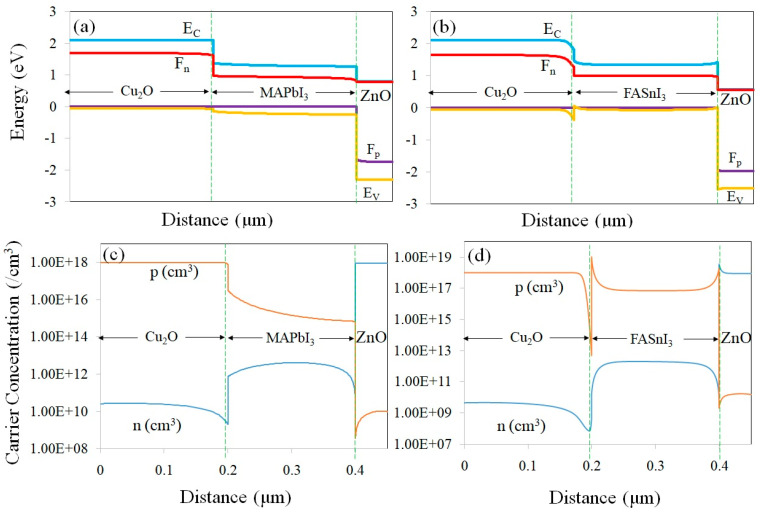
Simulation outcomes for the MAPbI_3_- (**a**,**c**) and FASnI_3_-based (**b**,**d**) perovskite solar cells under AM 1.5 spectrum.

In Figure 1c, the HOMO level of MAPbI_3_ is located at −5.54 eV, which is lower than that of the Cu_2_O (−5.37 eV), making sure a significant driving force is applied to the holes and resulting in their extraction from the perovskite layer and transfer into the HTL. Similarly, the lowest unoccupied molecular orbital (LUMO) levels of the perovskite and ZnO (electron transport layer, ETL) are located at −3.93 and −4.4 eV, respectively. This can provide a suitable path for electrons toward the ZnO nanolayer. In a device with a FASnI_3_ perovskite, for the best calibration, the initial carrier density of holes was assumed to be 7 × 10^16^ cm^−3^ [34]. The deficiencies at the ETL/perovskite and the perovskite/HTM interfaces are assumed to be single and/or neutral. The energy level diagram and carrier concentrations across the devices with MAPbI_3_ and FASnI_3_ perovskites under the AM 1.5 solar spectrum are presented in Figure 3. In the FASnI_3_-based PSCs, the HOMO level of FASnI_3_ is located at −4.93 eV, which is higher than that of Cu_2_O (−5.37 eV). This barrier for hole transporting increases the carrier recombination possibility at the FASnI_3_/Cu_2_O interface. Therefore, the V_OC_ decreases from 0.76 V to 0.54 V.

It can be seen from Figure 3a,b that the Fermi level of electrons (F_n_) is positioned under (~0.4 eV) the conduction band (E_C_) of the Cu_2_O, while it is very close to the E_C_ of ZnO (with only ~0.05 eV at the lower level). On the other hand, the hole Fermi level (F_p_) is located above the valence band (E_V_) of the ZnO and close to the E_V_ of Cu_2_O. Hence, it can be concluded that there is a remarkable minority carrier concentration in the contacts/interfaces. 

The electron (n) and hole (p) concentrations in the simulated devices with MAPbI_3_ and FASnI_3_ perovskites are depicted in Figure 3c and 3d, respectively. It can be observed that, near the ZnO/perovskite interface, the electron concentration (n) is high. Similarly, near the perovskite/Cu_2_O interface, the hole concentration (p) is high. It can be seen that the carrier concentrations in both devices are constant across the absorber layer. However, a sudden reduction in carriers near the contacts/interfaces could be because of the higher density of defects in these regions.

### 3.1. Effect of Perovskite Defect Density (N_t_) on the Device’s Performance

**Figure 4:**
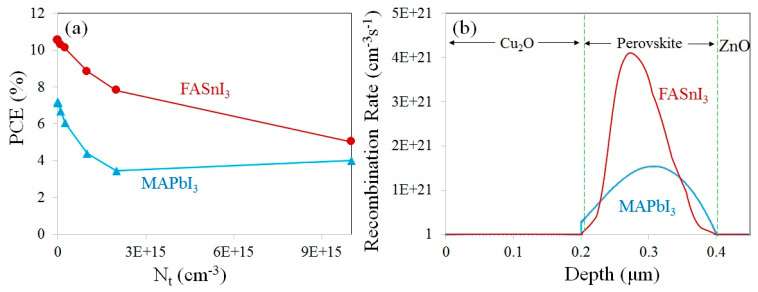
(**a**) Variation in the PCE of the cells due to the defect density of the absorber layer and (**b**) recombination rates along the depth of the multilayer structure of the MAPbI_3_- and FASnI_3_-based devices.

In this study, the perovskite defect density (N_t_) of the MAPbI_3_ and FASnI_3_ layers were set to 2.5 × 10^14^ and 2 × 10^15^ cm^−3^, respectively. This high value of N_t_ in the FASnI_3_ perovskite layer results in the trap-assisted Shockley–Read–Hall (SRH) recombination effect [35], which could be expressed as the following equation:

(1) $$R_{SRH}=\frac{np-n_{i}^{2}}{\tau_{p}\left(n+n_{1}\right)+\tau_{n}\left(p+p_{1}\right)}$$

where n and p are the electron and hole concentrations, τ_n,_ and τ_p_ are the electron and hole lifetimes, and n_i_ is the intrinsic carrier concentration. The n_1_ and p_1_ are defined by the following:

(2) $$n_{1}=N_{C}e^{\left(\frac{-E_{C}+E_{T}}{kT}\right)};\;p_{1}=N_{V}e^{\left(\frac{-E_{T}+E_{V}}{kT}\right)}\;$$

where N_C_ and N_V_ show the carrier densities, and E_C_ and E_V_ indicate the energy levels of the conduction and valence bands, respectively. In the SRH recombination model, the electrons in the CB (or the holes in VB) can recombine through trap sites. The open circuit electric potential is expressed by the following equation:

(3) $$V_{OC}=nV_{T}ln(1+\frac{I_{G}}{I_{0}})$$

where n, V_T_, I_G_, and I_0_ are the diode ideality factors, thermal electric potential, photogenerated current, and dark saturated current, respectively. Figure 4a presents the dependence of the PCE on the defect density (N_t_). variation in the perovskite layers (MAPbI_3_ and FASnI_3_). The recombination rate inside the perovskite layers is depicted in Figure 4b. Table 3 also shows the numerical results obtained for the PCE of the devices at various defect densities. 

By increasing the defect density in the absorber layer, the PCE of the cells, at first, decreased sharply and then showed a nearly saturated trend in both devices. Increasing the defects results in the enlargement of the series resistance of the device and, correspondingly, the reduction in the efficiency. In Figure 4b, it can be observed that the carrier recombination process through the FASnI_3_ absorber layer is 4.1 × 10^21^ cm^−3^s^−1^, which is higher than that of the MAPbI_3_ layer (1.54 × 10^21^ cm^−3^s^−1^). This higher recombination rate in FASnI_3_ decreases the V_OC_ of the cell. Thus, the V_OC_ of a cell with a FASnI_3_ absorber (0.54 V) is lower than that of a device with a MAPbI_3_ absorber layer (0.76 V). It should be noted that in Figure 2b, the J–V characteristic was found for the defect density of 2.5 × 10^14^ cm^−3^ for MAPbI_3_ and 2 × 10^15^ cm^−3^ for MAPbI_3_, resulting in efficiencies of 6.06% and 7.83%, respectively. Meanwhile, the thickness of the absorber layer was set to 200 nm in both devices.

### 3.2. Effect of the Perovskite Thickness on the Device’s Performance

**Figure 5:**
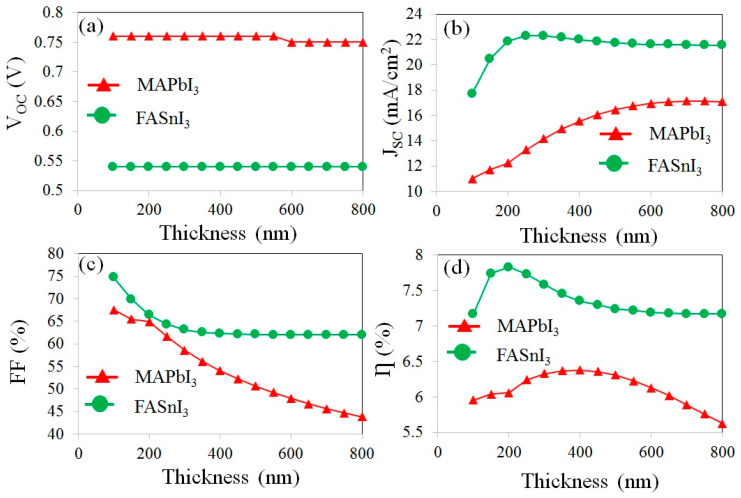
The effect of perovskite thickness on the various cell parameters, including (**a**) V_OC_, (**b**) J_SC_, (**c**) FF, and (**d**) PCE.

The influence of the perovskite thickness (varying in the range of 100–800 nm) on the photovoltaic properties of the cells was investigated as shown in Figure 5.

Figure 5a indicates that the variation in the perovskite thickness cannot affect the open circuit potential of the devices. This implies that maximum charge separation, as well as charge recombination, simultaneously occurred in the active layers.

In the device with FASnI_3_, the short circuit current density significantly increased with the increasing perovskite thickness and reached the maximum value of 22.29 mA/cm^2^ at the thickness of 300 nm. Then, it showed a slight decrease to reach the value of 21.55 mA/cm^2^ at the thickness of 800 nm, as exhibited in Figure 5b.

It was found that the FF drops continuously from 64.91% and 74.78% to 43.78% and 61.99% upon an increase in the MAPbI_3_ and FASnI_3_ thickness from 100 nm to 800 nm, respectively, as presented in Figure 5c. This decrease in the FF with an increasing thickness can be attributed to the increased recombination rate of the charge carriers inside the active layers. In the case of thicker absorber layers, the charge carriers do not have sufficient time to reach the desired energy band within the perovskite layer, resulting in premature recombination [20].

It can be seen from Figure 5d that the PCE is raised to 6.38% and 7.83% due to the increases in the thicknesses of the MAPbI_3_ and FASnI_3_ layers to 400 and 200 nm, respectively. The observed improvement in the PCE with an increase in the thickness of the perovskite layer can be attributed to the greater light absorption that occurs within the active layer. The PCE is unchanged after the thickness of the FASnI_3_ is increased 400 nm, while it is decreased slightly for the MAPbI_3_ after reaching the maximum value (6.38% at 400 nm). However, after reaching the thicknesses of 400 nm (of MAPbI_3_) and 200 nm (of FASnI_3_), the PCE decreased because of an increase in the recombination rate in the solar cells, which was induced by increasing the thickness. Therefore, the thicknesses of 400 and 200 nm were determined as optimum values for the MAPbI_3_ and FASnI_3_ layers, respectively. The decrease in the PCE after reaching the maximum value could be attributed to the growth in the recombination rate in a solar cell with a higher thickness.

**Figure 6:**
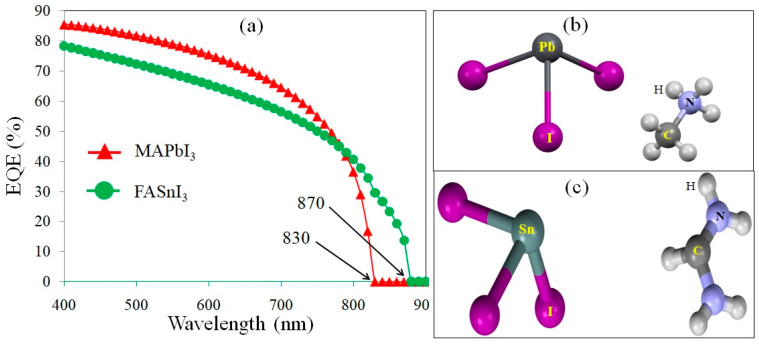
(**a**) The EQE outcomes of solar cells with the different perovskite absorber layers and partial views of the (**b**) MAPbI_3_ and (**c**) FASnI_3_ structures.

The external quantum efficiency (EQE) of the MAPbI_3_ and FASnI_3_-based solar cells exposed to the illumination of a solar simulator is shown in Figure 6a. The EQE curve in the visible wavelength region has a soft slope. A broad spectral response was obtained up to ~830 and 870 nm wavelengths, which correspond to 1.52 and 1.41 eV, i.e., the band gap values of the MAPbI_3_ and FASnI_3_ perovskites, respectively. The photons with energies lower than those of the band gaps of the absorber layers cannot be absorbed, causing vanished EQEs. Figure 6a shows that there is no significant difference in the EQE behaviors of the two perovskite layers at the short wavelength region (below 400 nm). However, for the MAGeI_3_-based device, the cutoff in the EQE trend presented a blue shift due to its larger band gap. Meanwhile, the integrated J_SC_ of the FASnI_3_-based cell was found to be ~35% higher than that of the MAPbI_3_-based one. A Cambridge Structural Database (CSD) [36] survey was searched to explore the structures of MAPbI_3_ and FASnI_3_ perovskites. The MAPbI_3_ and FASnI_3_ structures with CSD codes of FOLLIB [37] and WUFYEE [38] are shown in Figure 6b,c.

Devices with non-toxic absorbers (Pb-free perovskites) are friendly environment materials that have the potential for mass production. Although they show lower PV parameters, by improving the light absorption, this challenge may be solved. In addition, the low PV parameters observed can be attributed to a limited collection of carrier charges. Consequently, to enhance the collection of charge carriers, it is possible to decrease the thickness of the absorber layer [39]. Furthermore, low PV parameters reflect inadequate performance, as the performance of photovoltaic cells relies on the recombination kinetics of carriers within the device [40,41]. When the absorber thickness is too low, the absorption of light is also low, resulting in low PV parameters. Conversely, by increasing the thickness, there is a significant improvement in the power conversion efficiencies (PCEs) of the cells. However, once the thickness exceeds a specific amount, the growth of the PCE slows down. This is because if the absorber layer becomes too thick, the photogenerated carriers cannot be collected efficiently, as they must traverse the absorber before reaching the carrier collecting layers, leading to the quenching of charge carriers [42].

## 4. Conclusions

The FTO/ZnO/FASnI_3_/Cu_2_O/Au multilayer structure has been proposed as an environmentally friendly substitute for the MAPbI_3_-based one with ~30% better efficiency and 50% lower material consumption. The carrier concentrations remain constant throughout both devices, encompassing the absorber layer. However, a sudden decrease in carriers adjacent to the contacts/interfaces may be attributed to the elevated density of defects in these regions. More physical details, including the depth profile energy levels, charge carrier concentrations, and electron/hole recombination rate, have been examined within the multilayer structure. The values of FF exhibit a continuous decline from 64.9 and 74.78% to 43.78 and 61.99%, correspondingly, as the thicknesses of MAPbI_3_ and FASnI_3_ increase from 100 to 800 nm. A wide-ranging spectral response was acquired, encompassing approximately 830 to 870 nm wavelengths, which correspond to the band gap values of 1.52 and 1.41 eV for the MAPbI_3_ and FASnI_3_ perovskites, respectively. The results promise greater development of Pb-free perovskite solar cells with comparable and even better efficiencies compared to Pb-based ones, provided that suitable electron/hole transport layers can be designed along with the perovskite layer in the multilayer structures of solar cell devices.

## Figures and Tables

**Table 1:** The parameters considered for the simulation of the perovskite solar cells.

Parameters	ZnO	MAPbI_3_	FASnI_3_	Cu_2_O
Thickness (nm)	50	200	200	200
Energy band gap (eV)	3.3	1.52	1.41	2.1
e affinity (eV)	4.4	3.93	3.52	3.2
Dielectric constant	9	6.5	8.2	7.1
Conduction band density (cm^−3^)	1 × 10^18^	5 × 10^18^	1 × 10^18^	2 × 10^17^
Valance band density (cm^−3^)	1 × 10^19^	5 × 10^18^	1 × 10^18^	1 × 10^19^
Mobility of electrons (cm^2^/V.s)	100	2	22	200
Mobility of holes (cm^2^/V.s)	25	2	22	80
Density of donors N_D_ (cm^−3^)	1 × 10^17^	1 × 10^13^	7 × 10^16^	0
Density of acceptor N_A_ (cm^−3^)	0	0	0	1 × 10^18^
Density of defects N_t_ (cm^−3^)	1 × 10^14^	2 × 10^14^	2 × 10^15^	1 × 10^17^

**Table 2:** Comparison of the simulation results with experimental results reported in the literature.

Photovoltaic Parameter	Measurement (MAPbI_3_) [12]	Simulation (MAPbI_3_)	Simulation (FASnI_3_)
V_OC_ (V)	0.76	0.76	0.54
J_SC_ (mA/cm^2^)	12	12.26	21.83
FF (%)	63	64.91	66.37
Ƞ (%)	6.02	6.06	7.83

**Table 3:** The PCE variation due to the defect density of the absorber layer.

N_t_ (cm^−3^)	PCE (MAPbI_3_)	PCE (FASnI_3_)
1 × 10^11^	7.18	10.55
1 × 10^12^	7.18	10.55
1 × 10^13^	7.13	10.52
1 × 10^14^	6.68	10.32
2.5 × 10^14^	6.06	10.12
1 × 10^15^	4.39	8.85
2 × 10^15^	3.45	7.83
1 × 10^16^	4	5.03

## Data Availability

Data are contained within the article.
